# Bile Acids: The Contribution of the Gut Microbiota to Atherosclerosis

**DOI:** 10.31083/RCM44050

**Published:** 2026-01-13

**Authors:** Lijia Xu, Haojie Yang, Chaojie He, Honghong Zhang, Zhe Jiang, Yuhang Zhang, Kexin Luan, Huilin Hu

**Affiliations:** ^1^Department of Cardiology, Jiaxing University Master Degree Cultivation Base, Zhejiang Chinese Medical University, 310053 Hangzhou, Zhejiang, China; ^2^Department of Cardiology, The Affiliated Hospital of Jiaxing University, 314001 Jiaxing, Zhejiang, China

**Keywords:** gut microbiota, bile acids, atherosclerosis

## Abstract

Emerging evidence has implicated the gut microbiota in the pathogenesis and progression of numerous cardiovascular diseases. Atherosclerosis is a major pathological process that leads to many severe cardiovascular complications. Meanwhile, atherosclerosis patients may experience local and systemic inflammatory responses, with structural changes in the intestinal microbiota and increased mucosal permeability. Currently, the role of gut microbiota-derived metabolites in atherosclerosis pathology is of great concern. Relevant findings have highlighted the potential direct or indirect impacts of gut microbiota on the metabolic health of the host via the production of various metabolites. Thus, this review places an emphasis on bile acids (BAs), metabolites derived from and regulated by the gut microbiota. BAs can delay the pathological processes associated with atherosclerosis, underscoring the significance of these metabolites as an early marker for disease progression risk. In addition, we explore the potential of BA-related gut metabolites as novel therapeutic targets for atherosclerosis, and propose several promising directions for future research.

## 1. Introduction

Atherosclerosis is characterized by lipid accumulation and chronic inflammation involving medium- and large-sized arteries, accompanied by the proliferation of smooth muscle cells and fibromyosin [1]. It is a significant contributor to coronary atherosclerotic heart disease (CHD) and stroke. Currently, the prevalence of coronary heart disease, stroke, and other conditions has decreased, owing to enormous research and various therapeutic options. However, atherosclerosis remains a threat to human health. Pathologically, the debris of inflammatory, smooth muscle, and massive necrotic cells, as well as lipids, may accumulate and transform under the intima over a long period, eventually forming plaques. Simultaneously, angina pectoris may occur when the luminal blood flow is obstructed by ≥50% of the growing plaques, which may consequently trigger myocardial infarction (MI) due to their instability or rupture during subsequent exercise or under high pressure [2]. The current academic consensus recognizes hyperlipidemia, hypertension, and hyperglycemia as the major risk factors for atherosclerosis. These remain the primary targets of current therapeutic strategies. Therefore, the current major therapeutic options for atherosclerosis involve stabilizing the blood glucose and lipid levels, as well as controlling blood pressure. However, these measures remain insufficient to fully reduce the cardiovascular risk during primary and secondary prevention, underscoring the urgent need to identify novel therapeutic targets.

Documented evidence has emphasized the potential roles of certain intestinal microbiota and their metabolites in atherosclerosis development. Specifically, the dietary composition may disturb the intestinal microbiota structure, and the metabolites produced by certain bacterial strains may promote the progression of atherosclerosis [3]. Moreover, the intestinal microbiota may impact the host’s inflammatory response, altering the blood pressure and simultaneously accelerating atherosclerosis progression.

Among the numerous intestinal microbiota-derived metabolites, bile acids (BAs) emerge as particularly salient factors influencing atherosclerosis, and interventions targeting the BA receptor might delay plaque formation [4]. Hence, this review was designed to provide a comprehensive overview of the current state of research on the association between BAs and atherosclerosis, along with a critical evaluation of their potential for translation into novel therapeutic targets.

## 2. Disordered Intestinal Metabolism in Atherosclerosis

Acting as a major risk factor for numerous Cardiovascular Diseases (CVDs), atherosclerosis principally entails cholesterol accumulation and the recruitment of macrophages within the arterial wall, forming plaques [5]. Conversely, an inflammatory response constitutes the predominant mechanism in this context. Mounting evidence has confirmed that intestinal microbiota can modulate the inflammatory response and metabolite production by microbes, exerting a pathogenic role in atherosclerosis [6].

### 2.1 Intestinal Microbiota Dysbiosis

With an extremely complex microbial profile, the gastrointestinal tract contains an estimated 10^14^ microorganisms vital for human health. These may participate in organic matter digestion, production of various metabolites, modification of mucous membranes, pathogen defense, etc. Dysbiosis of intestinal microbiota represents a potential risk factor for various diseases, including atherosclerosis. Furthermore, atherosclerosis is associated with structural alterations in the intestinal microbiota. In a controlled investigation, an increase in the abundance of Enterobacteriaceae members and *Streptococcus* spp. was identified in the intestinal microbiome of atherosclerosis patients, accompanied by metabolic alterations that might promote disease progression [7]. A compositional assessment of atherosclerosis plaques indicated the presence of *Actinobacteria* spp. (including *Collinsella* spp.), *Chryseomonas* spp. and *Helicobacter* spp., as well as an increased abundance of the fungal genus *Aspergillus*, in individuals diagnosed with atherosclerosis compared to healthy individuals [8]. In summary, atherosclerosis advancement may be expedited owing to mutually reinforced alterations in the microbiota composition, induced by atherosclerosis and dysbiosis.

### 2.2 Altered Permeability in Intestinal Mucosa, Accompanied by Persistent Inflammation

Atherosclerosis is recognized as a pathological process entailing chronic inflammation [9], constituting a fundamental component, and pervading most stages of atherosclerosis progression [10]. It serves as a common basis for atherosclerosis physiology and pathology, which primarily manifests as a substantial surge in the plasma levels of various pro-inflammatory cytokines. Recent evidence posits that imbalances in intestinal microbiota may heighten mucosal permeability, accelerating inflammation [11].

Patients with unstable plaques might have an altered microbiota structure compared to those with stable plaques. Unstable plaques were also linked to reduced fecal levels of *Roseburia* and an augmented theoretical capacity of the microbiota to produce the pro-inflammatory peptidoglycan, along with a diminished synthesis of anti-inflammatory carotenoids [12]. Such alterations would facilitate the recruitment of inflammation-inducing factors by the lesion and accelerate plaque transformation, as demonstrated by numerous studies. The characterization of inflammatory vesicle complexes integrated immune receptor responses from a range of stimuli, including cytokines, infections, macromolecules, and reactive oxygen species [13]. These stimuli might enhance endotoxemia, in turn exacerbating inflammatory responses and oxidative stress within the vascular endothelium. Concurrently, the diminution of short-chain fatty acid-producing bacteria, such as *Roseburia*, might impede intestinal barrier function and augment wall permeability, thereby boosting the translocation of microbiota-derived metabolites, including lipopolysaccharides (LPS), into the bloodstream [14]. The levels of inflammatory markers (Ultrasensitive C-reactive protein, Interleukin [IL]-6, and IL-1β [IL-1β]) in the peripheral blood also increased. IL-6 and IL-1β are cytokines, immune system-related molecules that can regulate the functions of neighboring cells in a paracrine manner and induce vascular calcification [15]. Such a sustained inflammatory microenvironment may enhance foam cell formation, fibrous cap thinning, and matrix metalloproteinase activity, ultimately elevating plaque vulnerability.

In addition, the gut microbiota–immune system interaction is considered a critical component of the chronic inflammatory response observed in atherosclerosis [16]. Microbial metabolites can influence the differentiation and polarization of immune cells, such as a balance between the T helper cell subsets and the activation of macrophages, thereby shaping the inflammation-associated microenvironment of the vascular wall. Altered immune responses induced by gut microbial dysbiosis may thus represent a vital mechanistic link between the intestinal ecosystem and cardiovascular pathology [17].

Nonetheless, contemporary endeavors to treat atherosclerosis by suppressing the inflammatory response remain in the experimental phase, precluding the determination of a specific group of patients benefiting from anti-inflammatory therapy [18]. Consequently, the focus on the role of microbial metabolites in atherosclerosis has escalated.

### 2.3 Changes in Microbial Metabolites

A considerable body of empirical evidence corroborates the notion that gut microbiota exert systemic effects on the host as they enable the production of bioactive metabolites, such as short-chain fatty acids (SCFAs), BAs, and Trimethylamine-N-oxide (TMAO), which are particularly pronounced in the circulatory system [19]. In general, a majority of the bacterial metabolites typical of the human body promote well-being. However, disrupted gut microbiota can augment the precipitation of noxious metabolites, a phenomenon that may be pertinent to the pathological progression of atherosclerosis. Beyond traditional lipid metabolism, emerging microbe-derived metabolites have garnered increasing attention for their role in the pathogenesis and progression of atherosclerosis. Indole derivatives, such as indole-3-propionic acid (IPA) and indole-3-acetic acid (IAA), exert protective effects by activating the aryl hydrocarbon receptor and its related signaling pathways to modulate inflammation and endothelial function [20]. SCFAs, including acetate, propionate, and butyrate, act through GPR41/43 receptors and histone deacetylase (HDAC) inhibition to influence macrophage polarization, Treg differentiation, and vascular inflammatory responses, thereby enhancing plaque stability and maintaining metabolic homeostasis [21]. In contrast, recently, the microbe-derived metabolite imidazole propionate (ImP) was reported to be markedly elevated during the active stages of atherosclerosis, promoting inflammation and plaque progression via the imidazoline receptor (I1R), and is considered potentially pathogenic [22]. Moreover, BAs modulate cholesterol metabolism, inflammation-associated signaling, and endothelial function, thereby influencing the course of atherosclerosis and demonstrating a close association with disease prognosis.

## 3. Mechanisms of BA Metabolism

BAs are synthesized in the liver through a series of cytochrome P450 (CYP)-catalyzed cholesterol oxidation reactions [23]. The primary BA may further combine with varying ratios of taurine or glycine [24] to form the conjugated BA (CBA). The CBA subsequently exits the hepatocytes into the gall bladder following meals, along with the flow of bile into the intestines, where it fulfills a predominant role in facilitating the absorption of dietary lipids and vitamins by the small intestines [25]. The recycling mechanism of the human body is a sophisticated process, termed “enterohepatic recycling”. It involves the reabsorption of a majority of the plasma protein-bound BAs through a multifaceted transport mechanism. Specifically, it occurs due to active transport within the distal ileum along with passive absorption throughout the intestine. The reabsorbed BAs are then recirculated to the liver through the portal vein. Such an enterohepatic cycle occurs 4–12 times daily [26]. The colon may absorb ~5%–10% of the BAs, where they are either biotransformed by the gut microbiota or excreted [25]. During this process, the gut microbiota principally modulates the BA pool by mediating a series of enzyme-catalyzed reactions. These include a portal reaction facilitated by the hydrolysis of the C-24 N-acyl bonds by which BAs are bound to taurine or glycine, catalyzed by bile salt hydrolase (BSH) in the lower small intestine and proximal colon to yield the secondary BAs (SBAs) [27], thereby generating free BAs in the liver. Gram-positive bacteria (e.g., *Clostridium*, *Enterococcus*, *Bifidobacterium*, and *Lactobacillus*), which are among the most abundant intestinal microbiota, exhibit the greatest diversity regarding BSH distribution among numerous other species. Recently, BSH expression was noticed to be exclusive to the Anaplasma phylum among the Gram-negative bacteria [28]. Prior studies have substantiated the involvement of BAs in diverse biological processes, such as modulating lipid metabolism, regulating glucose metabolism, facilitating antimicrobial effects, and suppressing cholesterol synthesis [29].

Animal foods, particularly those comprising offal, eggs, select seafoods, and dairy products, contain notable cholesterol levels [30]. The conversion of cholesterol to BAs in the liver involves 17 enzymes, situated within the lysosomes, endoplasmic reticulum, mitochondria, and peroxisomes. Noticeably, cholesterol 7α-hydroxylase catalyzes the rate-limiting step of the process by which cholesterol is transformed into two predominant BAs in the liver. Cholic acid (CA) and deoxycholic acid (DCA) constitute the primary SBA components [31].

**Fig. 1.**
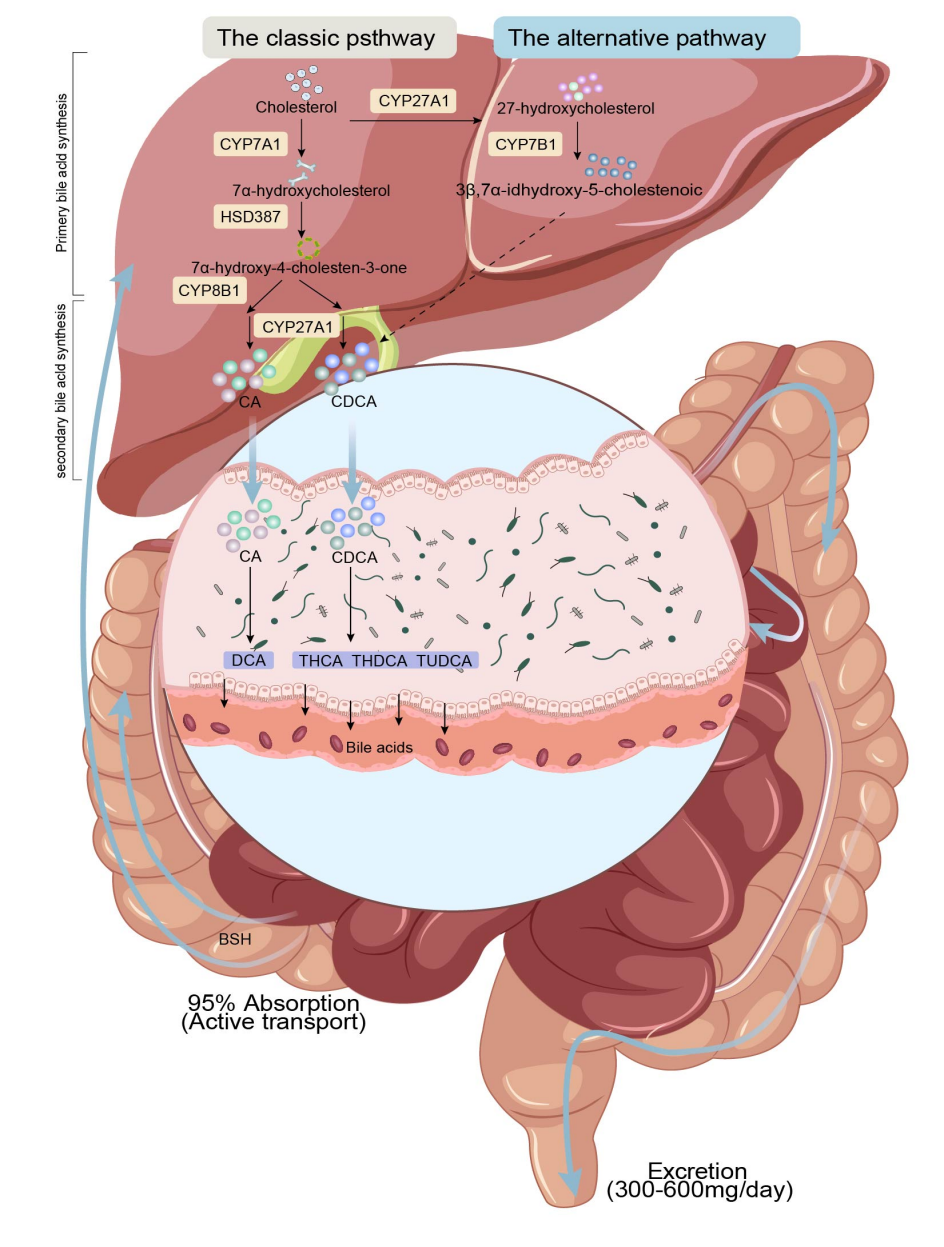
**BA metabolism and enterohepatic circulation**. In the liver, cholesterol undergoes a series of cytochrome P450 (CYP)-catalyzed reactions to form BAs, which may further facilitate the formation of CBA after integrating with taurine or glycine. CBA enters the intestines following the bile, where they form SBA. Most of the SBA dissociate under the catalytic action of BSH and return to the liver for enterohepatic recycling. A small fraction of SBA is excreted in the colon with feces. CDCA, Chenodeoxycholic acid; THCA, Trihydroxycholestane acid; THDCA, Taurohyodeoxycholic acid; TUDCA, Tauroursodeoxycholic acid; CA, Cholic acid; DCA, deoxycholic acid; BSH, bile salt hydrolase.

Atherosclerosis-triggered compositional alterations in fecal microbiota can modulate the circulating BA levels [32]. Human intestine-resident strains, including *Pasteurella smithii*, *Clostridium difficile*, and *Enterococcus faecium*, capable of synthesizing BSH have been identified. They can induce cholesterol accumulation within the macrophages (foam cell formation), promote the production of oxidized low-density lipoproteins by modulating BSH activity, and accelerate disease progression by augmenting the size and structural instability of atherosclerotic plaques. Similarly, atherosclerosis patients have exhibited an expanded proportion of certain bacterial strains within the gastrointestinal microbiota [33]. As a result, the alterations in gut microbiota may regulate BA levels by modulating BSH activity (Fig. 1).

Certain gut microbiota-derived metabolites, such as TMAO and ImP, influence BA levels. Evidence suggests that TMAO can suppress BA biosynthesis, thereby accelerating the development and progression of atherosclerosis, and is considered a critical mechanism underlying TMAO-induced atherogenesis [34]. ImP, a novel microbial metabolite, has recently been implicated in altering the BA pool composition and has been closely associated with diseases such as diabetes, atherosclerosis, and gastrointestinal inflammation [35].

It is imperative to acknowledge the significance of the genetic factors of intestinal microbiota in producing metabolites. The hypothesis that host genetics contributes substantially to the structure of the intestinal microbiota has been advanced under the premise that such a structural element can regulate immune-related pathways and metabolism-related phenotypes [36]. A strong association was identified between the ABO blood group system-associated genes and *FUT2* polymorphisms with several intestinal microbiota, principally the genera *Collinsella*, Bifidobacterium, and *Enterococcus faecalis* [37]. Meanwhile, a genome-wide analysis of the intestinal microbiota from 1812 subjects revealed that genetic factors accounted for ~10% of the variations in the microbiota [38]. Consequently, genetics is essential in BA production, given its indispensable ecological role in altering the structural composition of intestinal microbiota. Thus, it was hypothesized that genetics is also essential for BA production.

## 4. Diseases of BA Metabolism and Atherosclerosis

Several experiments have demonstrated that BAs can impede the progression of atherosclerosis in either a direct or indirect pattern (Table 1, Ref. [39, 40, 41, 42, 43, 44, 45]).

**Table 1.** **Main pathophysiological mechanisms of BAs in atherosclerosis**.

Authors (year)	Models/cells	Main findings	Ref.
Zhou *et al*. (2024)	Mice	BAs can modulate platelet function by regulating the Syk, Akt, Erk1/2, and syntaxin-11 pathways, which are associated with NCK1.	[39]
Qi *et al*. (2025)	Mice	DCA functions as an inhibitor of platelet activation due to its interaction with TGR5.	[40]
Guo *et al*. (2016)	Mouse bone marrow-derived macrophages and mice	BAs can suppress the formation of NLRP3 inflammatory vesicles by acting through the TGR5-cAMP-PKA signaling axis.	[41]
Gilles Lambert *et al*. (2003)	Mice	FXR is the primary regulator of normal cholesterol metabolism, and genetic alterations affecting FXR function have the potential to promote atherosclerosis.	[42]
Yoyo T Y Li *et al*. (2007)	RASMC	FXR exerts a regulatory effect on VSMC inflammation by curtailing the migration of these cells.	[43]
Li *et al*. (2008)	Rats	FXR enhances eNOS expression.	[44]
He *et al*. (2006)	Rats	FXR serves to diminish the manifestation of the vasoconstrictor endothelin (ET)-1.	[45]

FXR, Farnesoid-X-Receptor; TGR5, transmembrane G protein-coupled receptor-5; VSMC, vascular smooth muscle cell; eNOS, endothelial nitric oxide synthase.

### 4.1 BAs Modulate Platelet Function

Serum total BA (TBA) levels correlated directly with the extent of atherosclerotic lesions, with substantially reduced levels in patients with acute coronary syndrome (ACS), particularly in those with acute MI (AMI), than in the normal range [46]. Various SBAs, including DCA, lithocholic acid (LCA), and chenodeoxycholic acid (CDCA), can inactivate collagen-stimulated platelets determined by kinase analysis and by constructing a mouse atherosclerosis model. Furthermore, LCA and CDCA could suppress collagen, thrombin, U46619, and ADP-stimulated platelet aggregation in humans. DCA and CA impede collagen-stimulated platelet aggregation, while U46619 and ADP elicit such a response. Moreover, in another NCK1-knockout mouse model, BAs could act against platelet activation and aggregation, primarily by inhibiting the phosphorylation of NCK1-related Syk, Akt, ERK1/2, and syntaxin-11 [39], aligning with the documented anti-atherosclerosis effects of BAs. In addition, DCA could arrest platelet activation and thrombosis by interacting with the transmembrane G protein-coupled receptor-5 (TGR5) [40].

### 4.2 BAs Restrain an Inflammatory Response

**Fig. 2.**
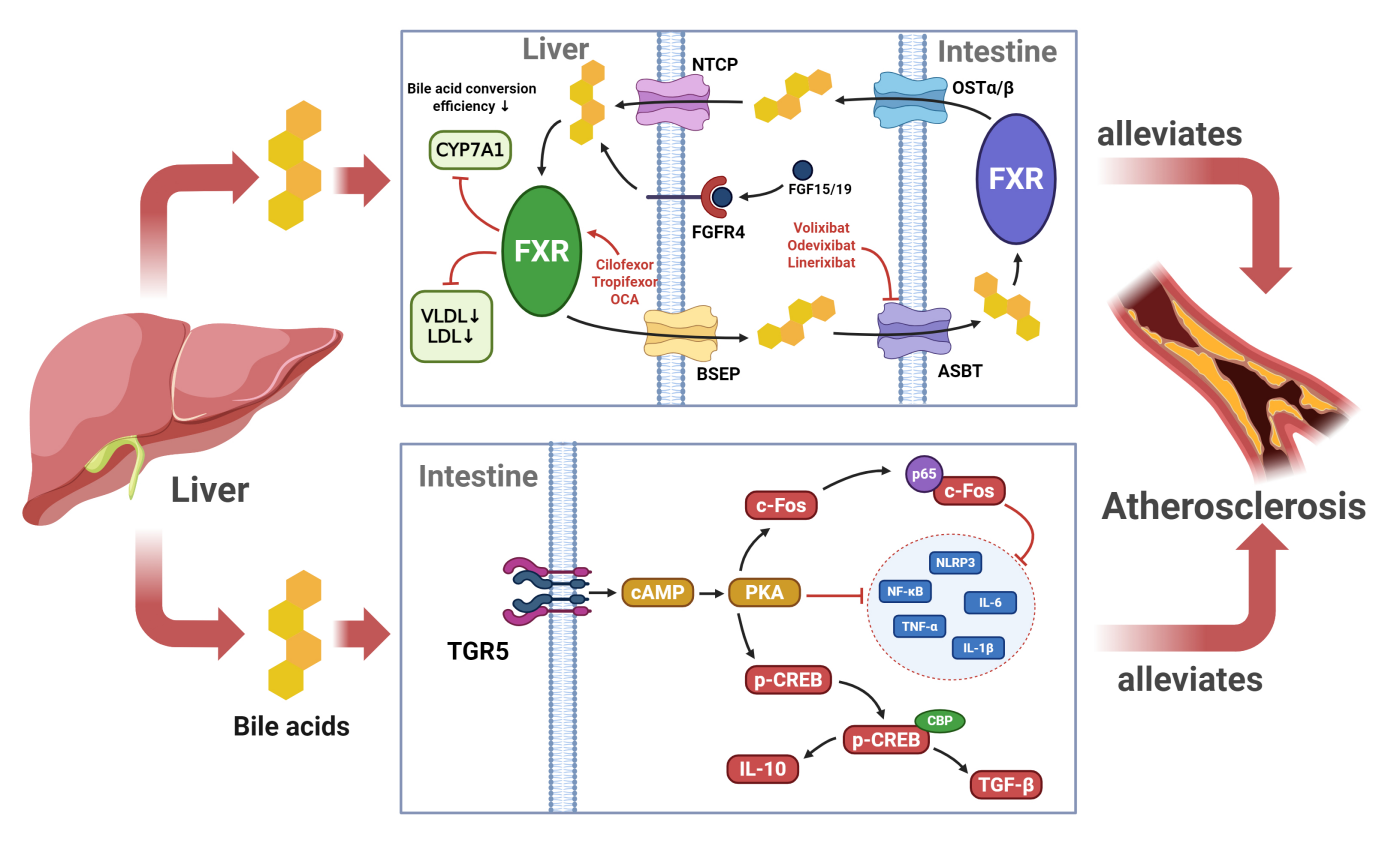
**Mechanisms underlying the inhibitory effect of BA on atherosclerosis progression**. BAs serve as an anti-inflammatory to suppress atherosclerosis progression by binding to TGR5 and FXR receptors to modulate the inflammatory response and maintain lipid homeostasis. NTCP, Sodium Taurocholate Cotransporting Polypeptide; OSTα/β, Organic Solute Transporter alpha/beta; BSEP, Bile Salt Export Pump; ASBT, Apical Sodium-Dependent Bile Acid Transporter.

Vascular inflammation is recognized as a pivotal pathological mechanism for atherosclerosis. Meanwhile, the nucleotide-binding, oligomerization, structural domain-like receptor family containing pyrin structural domain 3 (NLRP3) inflammatory vesicles assume a central role in the pathogenesis of vascular inflammation [47]. It is primarily linked to the upregulation of adhesion molecules facilitated by the enhanced processing and secretion of pro-inflammatory cytokines (e.g., IL-1β and IL-18). Vascular inflammation can be attributed to the upregulation of adhesion molecules [48]. In several animal models treated with mouse bone marrow-derived macrophages, BAs attenuated the activation of NLRP3 inflammatory vesicles and suppressed the release of IL-1β and IL-18. This effect appears to be achieved by inhibiting TGR5–cAMP–PKA signaling axis, eventually resulting in a hindered inflammatory response [41]. After binding to the TGR5 receptor, various BAs, including LCA, may activate protein kinase A (PKA) via the cAMP–PKA pathway, triggering the phosphorylation of Ser295 of NLRP3, thereby inhibiting it. Concurrently, PKA can induce the ubiquitination of NLRP3, further hampering its assembly and activation. Ultimately, this multifaceted mechanism inhibits inflammation and safeguards the vasculature (Fig. 2).

### 4.3 BAs Affect the Metabolism of Lipids

Lipid metabolism abnormalities and dyslipidemia have been recognized as risk factors for many cardiovascular diseases, generally presenting elevated plasma triglycerides (TGs) and low-density lipoprotein cholesterol (LDL-C). Subsequently, a massive deposition of these lipids within blood vessels induces local inflammation, vascular remodeling, and the formation of atherosclerotic plaques [10]. Farnesoid-X-Receptor (FXR) is the most intensively studied BA receptor [49], which is expressed in the pancreas, kidneys, vascular walls, and various other tissue types, along with enhanced expression in the gastrointestinal tract [50]. As a central mechanism by which FXR plays a role in metabolic regulation, besides constituting a key biological pathway for cholesterol catabolism, the FXR-mediated BA biosynthesis regulatory pathway can also reveal the dual roles of this nuclear receptor in lipid metabolism homeostasis through negative feedback. Specifically, it can regulate the efficiency of cholesterol–BA conversion by inhibiting the rate-limiting enzyme CYP7A1 [51], and also modulate BA conversion via signaling molecules such as FGF19/15 to maintain a dynamic homeostasis during systemic lipid metabolism [52] (Fig. 2). This pathophysiological mechanism serves as a theoretical basis for the targeted therapy of metabolic syndrome or associated diseases. In a controlled experiment, FXR knockout (FXR^-⁣/-^) mice with substantially increased plasma levels of apolipoprotein B-rich lipoproteins (i.e., non-HDL lipoproteins such as VLDL and LDL) were detected [42]. These observations demonstrated that FXR can reduce atherosclerosis-associated risk factors to a certain extent. In addition to controlling the metabolism-related risk factors for atherosclerosis, FXR is also expressed in different cell types of multiple blood vessels, including coronary arteries and aortic vascular smooth muscle cells (VSMCs) [53]. For instance, rat aortic smooth muscle cells were treated with the synthetic FXR ligands GW4064 or 6α-ethyl-goose DCA, which prompted apoptosis, down-regulated the IL-1β-induced expression of inducible nitric oxide synthase (iNOS) and cyclooxygenase-2 (COX-2), demonstrating its roles in curtailing VSMC inflammation and migration [43]. In the endothelial cells, FXR mediates the vascular tone by augmenting the expression of endothelial nitric oxide synthase (eNOS) [44] and the production of the vasodilator NO, while concomitantly diminishing the expression of the vasoconstrictor endothelin-1 (ET-1) [45], among other mechanisms. In summary, FXR activation by BAs evinces a certain degree of efficacy in retarding plaque formation. In addition, certain SBAs exert regulatory effects on cholesterol metabolism. In a clinical trial investigating the cholesterol-lowering effects of propionate (PA), 62 volunteers were randomly assigned in a 1:1 ratio to receive orally either the placebo or 500 mg PA twice daily for eight weeks. Blood samples were collected at the baseline and at the end of the intervention for metabolomics. The investigators observed that a reduction in cholesterol levels, particularly LDL-C, was negatively correlated with the plasma DCA levels, suggesting that DCA may favorably influence the cholesterol-lowering effects of PA [54]. Interestingly, in a controlled feeding study involving 11 volunteers who received either CDCA (15 mg/kg/day) or no BAs for 20 days, the oral administration of DCA impaired intestinal cholesterol absorption, reducing the plasma LDL-C levels [55]. These findings support the observations reported previously to a certain extent. Although animal studies have provided sufficient evidence that BAs can modulate lipid metabolism and absorption to exert protective effects during atherosclerosis, clinical trials remain relatively limited. Thus, the precise mechanisms are yet to be fully elucidated, warranting further in-depth investigations.

## 5. BAs as a Prognostic Marker of Adverse Events in Atherosclerosis

BAs signify a potentially auspicious risk indicator for cardiovascular events, which can forecast unfavorable outcomes in atherosclerosis patients to a certain extent.

### 5.1 CHD and MI

A substantial corpus of evidence confirms the robust anti-atherosclerotic effects of BAs, with CHD representing a particularly adverse event during the advanced stage. The serum and fecal BA levels in 45 CHD patients and 35 normal subjects were determined, revealing significantly lower total BA concentrations in CHD patients than in normal subjects. Thus, the absence of BAs in the serum served as a predictor for CHD [56]. Furthermore, the present study found that the administration of statins markedly elevated the serum BA levels in CHD patients, supporting their therapeutic value. Nevertheless, a study on the relationship between plasma acylcarnitines and BA levels with CHD incidence revealed a positive correlation between DCA and the documented risk of CHD, as suggested by Wu *et al*. [57]. This observation may be attributed to the premise that DCA, as a secondary BA, is predominantly antagonistic to FXR in the presence of CDCA [57]. This mechanism may explain the divergent associations among various BAs and CHD observed in this investigation.

A more exhaustive examination of TBA has predominantly occurred in the context of MI. A survey encompassing 7438 patients diagnosed with coronary artery disease ascertained the initial correlation between TBA and MI. Patients afflicted with MI exhibited a conspicuously diminished TBA in comparison to age- and sex-matched subjects without MI [58]. An experimental model involving BA administration in a MI mouse specimen exemplified the cardioprotective impacts of BAs. The experiment involved administering FXR-overexpressing, adipose tissue-derived mesenchymal stromal cells into the mouse myocardium through intramyocardial injection. Compared with the control group, the experimental group exhibited a substantial enhancement in the left ventricular ejection fraction, a concurrent reduction in the end-systolic left ventricular internal or diastolic diameters, and MI size. In particular, the experimental group mice displayed a higher survival rate in comparison to the control group [59]. Meanwhile, in a separate analytical study on ACS, a mediation analysis was employed to evaluate the serum TBA levels of patients in isolation, unveiling a negative correlation between the TBA concentration and coronary artery disease severity in patients experiencing AMI. In addition, the TBA concentration was independently associated with all-cause mortality and cardiac-related death risk. Conversely, a decline in the TBA levels might indicate an elevated risk of MI-related mortality [46].

### 5.2 Aneurysm

Atherosclerosis progression, accompanied by plaque hardening, may reduce the elasticity of the vessel wall [60]. This phenomenon is exemplified by the abdominal aorta among certain vessels characterized by elevated blood flow. The nonelastic vessel wall may not adequately counteract the wall tension engendered by the dynamic pulsation caused by blood flow, frequently precipitating localized verrucous dilatation, a condition clinically analogous to an aneurysm [61]. Despite their relatively limited prevalence relative to other cardiovascular conditions such as coronary heart disease and MI, aneurysms nevertheless pose a marked threat to atherosclerosis patients who are predisposed to rupture, triggering potentially severe consequences, including hemorrhage and high mortality. For instance, an experimental investigation was conducted by employing a 30-mouse abdominal aortic aneurysm (AAA) animal model to elucidate the functional role of BAs in aneurysms. Consequently, tauroursodeoxycholic acid (TUDCA) was found to curtail AAA formation in mice by impeding several signaling pathways [62]. While BAs harbor the potential to influence the prognosis of aneurysms, their efficacy as a diagnostic indicator for aneurysm development remains inconclusive.

### 5.3 Ischemic Stroke

Atherosclerosis of the intracranial large artery represents one of the most prevalent etiologies of ischemic stroke (IS) [63]. The underlying pathophysiological mechanisms encompass two primary pathways: atherosclerotic embolization within hemodynamically constricted vessels, culminating in suboptimal perfusion, thrombosis, and upstream embolism, in conjunction with plaque rupture or ulceration [64]. Building upon this hypothesis, atherosclerosis may serve as a potential pathophysiological IS mechanism, and IS prognosis may be predicted through the monitoring of atherosclerosis progression. In a randomized controlled trial (RCT), Monteiro-Cardoso *et al*. [65] observed that stroke-affected mice administered with CDCA had a significantly mitigated stroke infarct size, possibly due to the neuroprotective impacts of CDCA. Moreover, analogous observations were made by their research team in another prospective randomized case-control trial comprising 57 adult acute ischemic stroke patients and 54 age- and race-matched healthy individuals [65]. Another prospective epidemiological investigation of 7323 IS patients explored the correlation between serum TBA levels and the clinical outcomes of IS. The data confirmed a negative correlation between TBA levels at admission and post-admission prognosis. This finding substantiates the hypothesis that TBA serves as a prognostic IS biomarker [66].

Although BAs have considerable potential as prognostic indicators of various cardiovascular and cerebrovascular diseases, clinical trials remain relatively limited, with some findings being contradictory. For example, certain BAs, such as DCA, have demonstrated dual effects on atherosclerosis, raising concerns about their reliability over traditional lipid analyses. Moreover, BA levels are highly susceptible to dietary status, genetic background, infections, and dynamic changes in the gut microbiota, resulting in a lower stability than conventional biomarkers. Therefore, in-depth research and large-scale data validation are required to determine the feasibility of BAs as predictive indicators for atherosclerosis and related diseases.

## 6. Potential Therapeutic Targets in Atherosclerosis

**Fig. 3.**
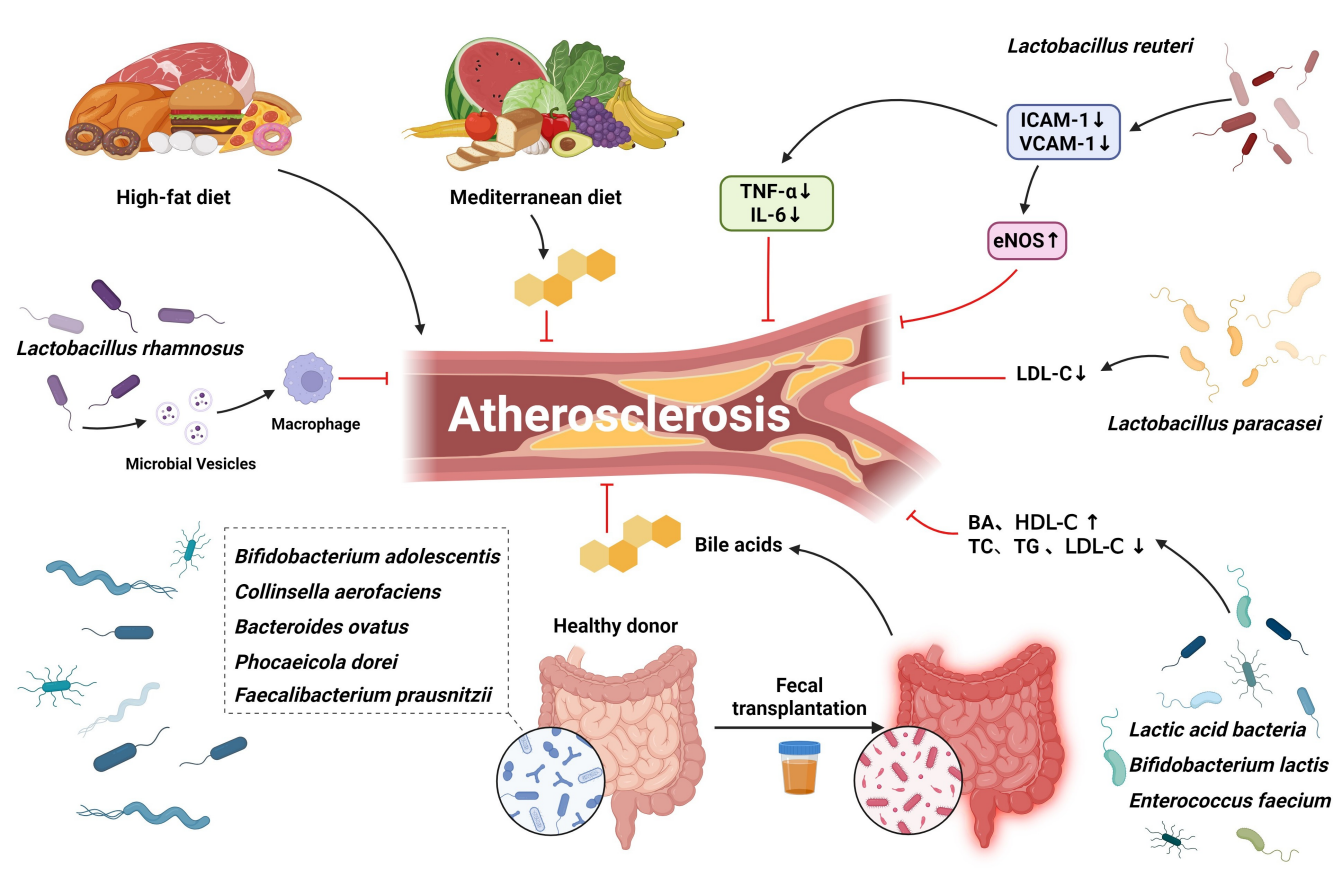
**Potential therapeutic targets for BAs-associated atherosclerosis in the gut**. The Mediterranean diet may promote the development of an optimal gut microbiota status and significantly reduce the prevalence of associated comorbidities, including atherosclerosis. Targeting gut microbiota, probiotics, prebiotics, and FMT may enhance Bas levels to some extent. Provides promising avenues for intervention in atherosclerosis. FMT, Fecal Microbiota Transplantation.

A comprehensive review of the studies conducted on diverse populations, as well as clinical observations, reveals a correlation between gut microbiota dysbiosis and circulating BA levels, with susceptibility to atherosclerosis, highlighting BA-associated intestinal microbiota-derived metabolites as promising therapeutic targets for atherosclerosis (Table 2, Ref. [67, 68, 69, 70, 71, 72, 73, 74]; Fig. 3).

**Table 2.** **Representative research on the potential treatment of atherosclerosis**.

Treatments	Interventions	Patients/models	Main findings	Ref.
Diet	High-fat diet + BA	Mice	The supplementation of a high-fat diet with a certain number of BAs reduces the risk of atherosclerosis.	[67]
	Mediterranean diet	Metabolic Syndrome	The Mediterranean diet interferes with BA metabolism.	[69]
	Mediterranean diet	CHD	Mediterranean diet superior to traditional low-fat diet in the prevention of major adverse cardiovascular events in atherosclerosis patients.	[68]
Probiotics	MVs isolated from *Lactobacillus rhamnosus*	Mice	Probiotic membrane vesicles (MVs) induce atherosclerotic plaque regression by modulating foamy macrophages.	[70]
	*Limosilactobacillus reuteri*	Mice	*Lactobacillus Royale* alleviates vascular endothelial dysfunction.	[71]
	*Lactobacillus vaginalis FN3*, *Bifidobacterium animalis subsp*. *Lactis F1–7*, *Enterococcus faecium WEFA23*	Mice	Probiotics inactivate the FXR-FGF15 signaling pathway to elevate intestinal progenitor BA levels.	[72]
Prebiotics	Dietary fiber	Mice	Dietary fiber protects against MI.	[73]
FMT	Intestinal microbiota from WT mice	Mice	FMT for restoration of gut microbial homeostasis may be an effective therapeutic strategy in atherosclerosis.	[74]

### 6.1 Dietary Intervention 

Plasma BA levels correlate robustly with dietary habits, underscoring the pivotal role of nutrition in the etiology of numerous cardiovascular diseases and the urgency for addressing this critical issue during medical inquiry. From the synthesis of BAs to their biotransformation in the gastrointestinal tract, numerous components exist that are prone to diet-based interventions. Given the great fluctuation in intestinal microbiota composition throughout human life [75], the application of such interventions precipitates more rapid alterations in the levels of the associated metabolites [76]. Western diets, which frequently comprise elevated saturated fatty acid levels, represent a remarkable contributor to the prevalence of gut microbial dysbiosis, a condition characterized by the altered composition of intestinal microbiota, and a concomitant suppression of BA secretion. Several studies have revealed a positive correlation between diet and the escalated risk of inflammation, particularly in the context of ulcerative colitis and Crohn’s disease, prompting the recommendation of dietary modifications as a potential therapeutic approach under such conditions [77]. Conversely, in a murine model on a high-fat diet, the incorporation of a specific number of BAs impeded weight gain and substantially diminished the prevalence of associated comorbidities, encompassing atherosclerosis [67]. However, the oral administration of BAs is exclusive to patients diagnosed with genetic defects resulting in a deficiency in primary BA synthesis [78]. Consequently, in the realm of atherosclerosis, it represents a salient contemporary issue regarding the investigation of the therapeutic potential of enhancing BA levels through dietary interventions. In similar inquiries on heart failure (HF), the Mediterranean diet—known for its numerous health benefits—has been endorsed for HF patients as it can foster an optimal composition of intestinal microbiota and curtail the prevalence of HF by ≤70% [68]. It is distinguished by an abundant use of fruits, vegetables, nuts, and whole grains, along with a moderate intake of meat, eggs, and sugar. Such a diet plan has been confirmed to markedly impact BA metabolism in a cross-over RCT comprising 44 patients with metabolic syndrome [69]. In a randomized controlled trial by Benjamin Seethaler *et al*. [79], the Mediterranean diet was shown to exert its primary effect on SBA levels, a change strongly linked to enhanced intestinal barrier function. In another large, multicenter, randomized, controlled, parallel-group clinical trial (the Prevención con Dieta Mediterránea [PREDIMED]) study, Montserrat Fitó *et al*. [80] demonstrated that adherence to a Mediterranean diet modulates both the composition and levels of BAs, significantly reduces LDL levels, and exerts protective effects against atherosclerosis. Based on these findings, the Mediterranean diet has been recommended as a powerful tool for atherosclerosis prevention [80]. Similarly, an RCT encompassing 1002 subjects examined the feasibility of the Mediterranean nutrition plan as a therapeutic intervention for CHD patients; such a diet exhibited a marked superiority over conventional low-fat diets in terms of its efficacy in averting major adverse cardiovascular events related to atherosclerosis [68]. Furthermore, the Mediterranean diet has demonstrated advantages in the prevention of atherosclerosis. In a single-center RCT (the CORDIOPREV study), adherence to a Mediterranean diet delayed the progression of coronary atherosclerosis, with the investigators recommending the incorporation of this dietary pattern into secondary prevention strategies for cardiovascular disease [81]. Similarly, a recent retrospective study involving 3532 participants aged 60 years from the Swedish Visualization of Asymptomatic Atherosclerotic Disease for Optimum Cardiovascular Prevention (VIPVIZA) trial demonstrated that following a Mediterranean diet was associated with a lower incidence of subclinical carotid atherosclerosis, thereby reinforcing the Mediterranean diet as a potential preventive strategy against diverse cardiovascular diseases [82].

However, the specific impact of the Mediterranean diet on BAs and its mechanistic link to anti-atherosclerotic effects remains largely unexplored. Beyond its well-established benefits, specific dietary components such as fibers, polyphenols, and omega-3 fatty acids can modulate BA metabolism by regulating the gut microbiota, thereby influencing the development of atherosclerosis [83]. For example, dietary fibers can be fermented by gut microbes to produce SCFAs, which not only enhance intestinal barrier integrity but also indirectly affect BA synthesis and transformation, subsequently modulating the FXR and TGR5 signaling pathways [84]. Polyphenols, conversely, may alter the composition and proportion of the BA pool by suppressing the growth of pathogenic microbes while promoting the proliferation of beneficial bacteria, ultimately influencing inflammatory responses and cholesterol metabolism [85]. Collectively, these findings suggest that dietary components regulate the “gut microbiota–bile acid–signaling axis”, thereby playing a crucial role in maintaining metabolic homeostasis and reducing the risk of atherosclerosis.

A nutritious diet refers to the most straightforward and economical approach to address and manage atherosclerosis by positively affecting the composition of the gut microbiota. Nevertheless, there is a paucity of research on effective interventions for atherosclerosis via enhancing BA levels through dietary adjustment. Promising avenues for future exploration involve the testing of BAs, cholesterol, LDLs, HDLs, and other markers to tailor dietary regimens to individual patients.

### 6.2 Probiotics and Prebiotics

Probiotics comprise live microorganisms that promote gastrointestinal health, which can modify the intestinal microbial composition and preserve homeostasis, eventually enhancing human health. They may facilitate the attenuation of atherosclerotic plaque softening, improvement in lipid parameters, curtailment of inflammatory responses, etc., eventually ameliorating the atherosclerosis-related parameters. For illustration, probiotic membrane vesicles isolated from *Lactobacillus rhamnosus* could induce atherosclerotic plaque regression by modulating foamy macrophages and exert a vasoprotective effect in mice with atherosclerosis [70]. Similarly, *Lactobacillus reuteri*, with anti-oxidant and anti-inflammatory properties, substantially mitigated the impacts of cell adhesion molecules (e.g., intercellular adhesion molecule-1 [ICAM-1]) and blood lipid levels [71]. *Lactis* F1-7 and *Enterococcus faecalis* WEFA23 regulated CYP7A1 expression in hyperlipidemic mice by inhibiting the FXR–FGF15 pathway, ultimately elevating the intestinal BA and HDL-C levels, while concomitantly suppressing serum TC, TG, and LDL-C levels [72]. Furthermore, substantial investigations involving animal models have demonstrated the capacity of probiotics to postpone the onset of atherosclerosis and safeguard blood vessels through diverse mechanisms. To validate their effectiveness in a clinical context, a 4-week interventional experiment on studying the effects of probiotics in patients with severe atherosclerosis was conducted, with a noteworthy enhancement in the abundance of beneficial gut strains, such as the *Lactobacillus plantarum* probiotic strain (DSM 9843) group, compared to the placebo group. The probiotic group exhibited a conspicuous augmentation in the diversity of beneficial intestinal strains [86]. Additionally, a prospective RCT in hyperlipidemic patients with comorbid early-onset atherosclerosis demonstrated that *Lactobacillus paracasei* TISTR 2593 considerably diminished LDL-C levels, contributing to anti-atherosclerosis by regulating the LDL-C levels [87]. Collectively, probiotic-based strategies continue to evince ambitious promise in the treatment of atherosclerosis, despite the dearth of studies on the impacts of probiotics in this group.

Another strategy to modulate gut homeostasis entails prebiotic administration. Prebiotics consist of indigestible carbohydrates that selectively regulate the proliferation, composition, and functionality of the gut microbiota [88], thereby improving host health to a certain extent. A series of experiments discovered that prebiotics enhanced intestinal permeability and alleviated metabolic endotoxemia, while concomitantly diminishing inflammatory responses in an obese mouse model [89]. Furthermore, in another study, MI-induced mice were administered prebiotics composed of dietary fibers, thereby remarkably improving cardiac function, diminishing infarct size, and preventing adverse remodeling post-MI. Subsequent metabolomics revealed that high-fiber diets elevated the BA levels, underscoring the protective role of dietary fibers in MI [73]. Extensive research on the prebiotic inulin has revealed its capacity to stimulate the proliferation of beneficial intestinal microbiota, optimize their diversity, and functionality. Concurrently, it can facilitate the mitigation of probiotics-related adverse effects [90], which, however, show no effect on ameliorating atherosclerosis. In an RCT on mice with hypercholesterolemia, inulin did not demonstrate any remarkable impact on plasma cholesterol levels. Additionally, it was impossible to prevent or delay atherosclerosis progression, despite increasing the proportion of specific bacterial genera and elevating the cecal SCFA levels [91]. Following previous inquiries, another investigation discovered that inulin can trigger a substantial escalation in total plasma cholesterol levels, while concomitantly expediting the progression of atherosclerosis in hypercholesterolemic E3L mice [92]. Cautiously, diverse prebiotics elicited contrasting effects on atherosclerosis, requiring further research to decipher the precise underlying etiologies and mechanisms.

Although some preliminary studies have demonstrated the theoretical anti-atherosclerotic potential of probiotics and prebiotics, clinical evidence and practical applications remain limited, and their effects are still debated. For example, animal experiments have shown that inulin may exert protective effects against atherosclerosis by promoting the production of SCFAs, thereby improving lipid metabolism and the inflammatory status [93]. However, inulin may exacerbate the progression of atherosclerosis in mouse models on a high-fat diet, possibly due to excessive fermentation leading to the accumulation of metabolites such as SBAs or other microbial byproducts, as well as causing microbiota dysbiosis [92]. Thus, the effects of prebiotics may be double-edged, influenced by host metabolic background, gut microbial composition, and dosage. Future research should aim to identify specific prebiotic formulations that provide consistent cardiovascular benefits across populations. For instance, galacto-oligosaccharides and fructo-oligosaccharides in certain clinical studies have shown the potential to improve lipid profiles and inflammatory markers [94]. These findings suggest that interventions targeting atherosclerosis should consider individual selection of prebiotic types and dosages based on host microbial characteristics, to maximize therapeutic benefits while minimizing potential risks.

### 6.3 Fecal Microbiota Transplantation (FMT)

FMT has recently been established as an available therapeutic option for diseases related to intestinal ecological dysregulation, mainly by transplanting intestinal microorganisms from healthy individuals to patients with certain intestinal disorders. FMT can combat enteric conditions such as *Clostridioides difficile* infection and inflammatory bowel syndrome [95], as well as several nonintestinal conditions such as neurological conditions [96], autism [97], obesity [98], and diabetes mellitus [99]. FMT was performed on patients with metabolic syndrome, resulting in the increased diversity of fecal microbiota, prominently associated with improved insulin sensitivity [100]. These positive features were confirmed in a subsequent RCT [101], illustrating the therapeutic potential of FMT against metabolic syndrome. Furthermore, in a secondary analysis of this double-blind RCT, obese patients with metabolic disorders were treated with FMT capsules donated by a lean donor. Consequently, after the successful colonization of the recipients with FMT, the evolution of glucose intolerance was delayed, as described previously. Additionally, the increase in BA metabolism induced by gut bacteria, comprising *Bifidobacterium adolescentum*, *Collinsella aerofaciens*, *Anabaena ovale*, *Phocaeicola dorei*, and *Faecalibacterium prausnitzii,* was positively associated with intestinal microbiota-derived BAs, which might be a potential therapeutic option for metabolic syndrome [102].

In light of these findings, FMT appears to possess therapeutic potential against atherosclerosis. In a seminal experiment, FMT was performed on atherosclerosis-prone mice, termed C1q/TNF-related protein 9 knockout (CTRP9-KO) mice. Transplantation of fecal matter from wild-type mice diminished atherosclerotic lesions in the carotid arteries of the CTRP9-KO mice, retarding the occurrence of atherosclerosis. Building upon these revelations, subsequent experiments by the same research group sought to promote atherosclerosis progression in wild-type mice through fecal transplantation from CTRP9-KO mice. In successive experiments, it was observed that the lesions promoted atherosclerosis propagation in the wild-type mice. The investigators concluded that the restoration of homeostasis in intestinal microbiota by FMT may serve as an effective strategy for atherosclerosis [74].

Indeed, FMT is efficient for treating atherosclerosis in animal models. Its clinical application still faces significant ethical and practical challenges. First, donor variability is a major limiting factor influencing the efficacy of FMT, as differences in the composition and functions of donor-derived microbiota may lead to substantial heterogeneity in clinical outcomes among the recipients [103]. Second, FMT carries potential safety concerns, including the transmission of pathogens, the transfer of antimicrobial resistance-associated genes, and the risk of adverse immune reactions. The U.S. Food and Drug Administration reported a case of death due to severe bacterial infection post-FMT, and issued a safety warning in 2019 [104]. In addition, FMT lacks long-term follow-up clinical evidence, and its efficacy and safety in cardiovascular diseases have not yet been systematically validated [105]. Consequently, while FMT shows promise in potentially transforming the therapeutic approach for numerous cardiovascular diseases, including atherosclerosis, further rigorous investigations are imperative to ascertain its practical clinical significance, particularly in addressing safety concerns. Moreover, before FMT can be considered as an intervention for atherosclerosis, it is essential to establish stringent donor screening criteria and long-term safety evaluation systems, as well as to explore standardized and controllable alternative approaches (such as synthetic microbial consortia or specific functional probiotic combinations) to minimize potential risks and enhance clinical feasibility.

## 7. Future Prospects

The structure of the intestinal microbiota or related metabolites has exhibited a profound association with atherosclerosis, as demonstrated by multiple human clinical trials. However, a majority of these analyses consist of typical correlations, hindering the establishment of a causal relationship between atherosclerosis and intestinal microbiota. Consequently, future research should prioritize prospective studies integrating pertinent metabolomic data, clinical data, and dietary factors to investigate the causal relationship among changes in intestinal microbiota.

In essence, the benefits of BAs for atherosclerosis can be categorized into two distinct clinical applications. The role of BAs in predicting atherosclerosis prognosis and as a potential therapeutic target involves their production in the liver, which plays a functional role through the transformation of intestinal microbiota. Variations in BA levels potentially mirror modifications in the composition of intestinal microbiota and the extent of ecological dysfunction. BAs also exhibit correlations with cholesterol and other lipids, encompassing LDLs, HDLs, and related compounds, all of which are of particular relevance to understanding the occurrence of atherosclerosis and patient survival. Hence, BAs serve as a valuable tool for healthcare professionals, assisting in the identification of high-risk patients while ensuring prompt follow-up testing and intervention. Notably, these factors are also intimately associated with adverse events and survival rates in atherosclerosis patients. Furthermore, treatment outcomes may be improved by the clinical screening of high-risk patients, along with follow-up testing and timely intervention. Nevertheless, there is a paucity of relevant studies; moreover, those available are mainly on Caucasians from Europe and the United States, with an absence of ethnic diversity. Thus, to ascertain the viability of BAs as a prognostic monitoring indicator and therapeutic target for atherosclerosis, there is an urgent need for further cohort analyses encompassing a more extensive array of ethnic types. In light of the preceding findings, pertinent interventions are feasible to elevate the levels of plasma BAs to impede deterioration in atherosclerosis.

Future research should also aim to define the optimal BA-targeted intervention strategies for the different subtypes of atherosclerosis, such as stable vs. unstable plaques. It is crucial to further elucidate whether distinct BA receptor pathways (e.g., FXR, TGR5) or specific microbiota-derived BA profiles exert beneficial or detrimental effects in varied patient populations. In addition, it should be recognized that various intervention modalities—including dietary modification, probiotics, pharmacological agents, or fecal microbiota transplantation—may yield heterogeneous outcomes across individuals, underscoring the necessity of developing personalized therapeutic approaches.

Additional research leveraging corresponding methodologies is pending, despite the presence of prior investigations that have employed gut microbial genome sequencing to identify the genera associated with BAs, TMAO, SCFA, and analogous metabolites. Nevertheless, there remains a substantial gap between theoretical underpinnings and their practical implementation in clinical settings. Conversely, the utilization of metabolites derived from gut microbes in blood, urine, and feces to direct targeted interventions demonstrates an enhanced clinical transformative value. Overall, these efforts can provide new directions for identifying novel therapeutic targets, optimizing risk stratification, and developing personalized intervention strategies, thereby reducing the disease burden associated with atherosclerosis.

## 8. Conclusion

To sum up, intestinal microbiota correlates intimately with various forms of cardiovascular diseases. BAs, one of the byproducts of intestinal microbiota-catalyzed biotransformation, have emerged as promising candidates for elucidating the mechanisms underlying the eliciting of atherosclerosis by gut microbiota. Correspondingly, these observations offer novel concepts for the subsequent development of interventions targeting the intestinal microbiota for atherosclerosis treatment. From a clinical perspective, these findings highlight the potential value of gut microbiota and BA-related biomarkers in risk stratification, prevention, and management of atherosclerosis. They also open new avenues for therapeutic intervention, examples of which include dietary modifications, probiotics, prebiotics, and FMT. The modulation of intestinal microbiota composition and the targeting of elevated BA levels represent promising avenues for the treatment of atherosclerosis. For researchers, these advances underscore the necessity of conducting in-depth mechanistic studies, large-scale multiethnic cohort investigations, and well-designed clinical trials to validate causal relationships and evaluate therapeutic efficacy. In the future, additional microbiota-derived metabolites associated with atherosclerosis are expected to be identified, which will further advance the development of safer and more effective microbiota- and BA-targeted therapeutic strategies, ultimately helping to reduce the global burden of cardiovascular diseases.

## References

[1] Zhu Y, Xian X, Wang Z, Bi Y, Chen Q, Han X (2018). Research Progress on the Relationship between Atherosclerosis and Inflammation. *Biomolecules*.

[2] Björkegren JLM, Lusis AJ (2022). Atherosclerosis: Recent developments. *Cell*.

[3] Yang S, Li X, Yang F, Zhao R, Pan X, Liang J (2019). Gut Microbiota-Dependent Marker TMAO in Promoting Cardiovascular Disease: Inflammation Mechanism, Clinical Prognostic, and Potential as a Therapeutic Target. *Frontiers in Pharmacology*.

[4] Verhaar BJH, Prodan A, Nieuwdorp M, Muller M (2020). Gut Microbiota in Hypertension and Atherosclerosis: A Review. *Nutrients*.

[5] Gui T, Shimokado A, Sun Y, Akasaka T, Muragaki Y (2012). Diverse roles of macrophages in atherosclerosis: from inflammatory biology to biomarker discovery. *Mediators of Inflammation*.

[6] Kasahara K, Tanoue T, Yamashita T, Yodoi K, Matsumoto T, Emoto T (2017). Commensal bacteria at the crossroad between cholesterol homeostasis and chronic inflammation in atherosclerosis. *Journal of Lipid Research*.

[7] Liu H, Chen X, Hu X, Niu H, Tian R, Wang H (2019). Alterations in the gut microbiome and metabolism with coronary artery disease severity. *Microbiome*.

[8] Karlsson FH, Fåk F, Nookaew I, Tremaroli V, Fagerberg B, Petranovic D (2012). Symptomatic atherosclerosis is associated with an altered gut metagenome. *Nature Communications*.

[9] Ross R (1999). Atherosclerosis–an inflammatory disease. *The New England Journal of Medicine*.

[10] Libby P, Ridker PM, Hansson GK (2011). Progress and challenges in translating the biology of atherosclerosis. *Nature*.

[11] Cox AJ, West NP, Cripps AW (2015). Obesity, inflammation, and the gut microbiota. *The Lancet. Diabetes & Endocrinology*.

[12] Anto L, Blesso CN (2022). Interplay between diet, the gut microbiome, and atherosclerosis: Role of dysbiosis and microbial metabolites on inflammation and disordered lipid metabolism. *The Journal of Nutritional Biochemistry*.

[13] Duewell P, Kono H, Rayner KJ, Sirois CM, Vladimer G, Bauernfeind FG (2010). NLRP3 inflammasomes are required for atherogenesis and activated by cholesterol crystals. *Nature*.

[14] Sandek A, Anker SD, von Haehling S (2009). The gut and intestinal bacteria in chronic heart failure. *Current Drug Metabolism*.

[15] Kapustin AN, Chatrou MLL, Drozdov I, Zheng Y, Davidson SM, Soong D (2015). Vascular smooth muscle cell calcification is mediated by regulated exosome secretion. *Circulation Research*.

[16] I Fernández-Avila A, Gutiérrez-Ibanes E, Martín de Miguel I, Sanz-Ruiz R, Gabaldón Á, Fernández-Avilés F (2024). One-year longitudinal changes of peripheral CD4+ T-lymphocyte counts, gut microbiome, and plaque vulnerability after an acute coronary syndrome. *International Journal of Cardiology. Heart & Vasculature*.

[17] Saigusa R, Winkels H, Ley K (2020). T cell subsets and functions in atherosclerosis. *Nature Reviews. Cardiology*.

[18] Raggi P, Genest J, Giles JT, Rayner KJ, Dwivedi G, Beanlands RS (2018). Role of inflammation in the pathogenesis of atherosclerosis and therapeutic interventions. *Atherosclerosis*.

[19] Tang WHW, Li DY, Hazen SL (2019). Dietary metabolism, the gut microbiome, and heart failure. *Nature Reviews. Cardiology*.

[20] Luo Z, Yang L, Zhu T, Fan F, Wang X, Liu Y (2024). Aucubin ameliorates atherosclerosis by modulating tryptophan metabolism and inhibiting endothelial-mesenchymal transitions via gut microbiota regulation. *Phytomedicine*.

[21] Li M, van Esch BCAM, Henricks PAJ, Folkerts G, Garssen J (2018). The Anti-inflammatory Effects of Short Chain Fatty Acids on Lipopolysaccharide- or Tumor Necrosis Factor α-Stimulated Endothelial Cells via Activation of GPR41/43 and Inhibition of HDACs. *Frontiers in Pharmacology*.

[22] Mastrangelo A, Robles-Vera I, Mañanes D, Galán M, Femenía-Muiña M, Redondo-Urzainqui A (2025). Imidazole propionate is a driver and therapeutic target in atherosclerosis. *Nature*.

[23] Axelson M, Ellis E, Mörk B, Garmark K, Abrahamsson A, Björkhem I (2000). Bile acid synthesis in cultured human hepatocytes: support for an alternative biosynthetic pathway to cholic acid. *Hepatology*.

[24] Johnson MR, Barnes S, Kwakye JB, Diasio RB (1991). Purification and characterization of bile acid-CoA: amino acid N-acyltransferase from human liver. *The Journal of Biological Chemistry*.

[25] Gonzalez FJ (2012). Nuclear receptor control of enterohepatic circulation. *Comprehensive Physiology*.

[26] Chiang JYL (2013). Bile acid metabolism and signaling. *Comprehensive Physiology*.

[27] Ridlon JM, Kang DJ, Hylemon PB (2006). Bile salt biotransformations by human intestinal bacteria. *Journal of Lipid Research*.

[28] Jones BV, Begley M, Hill C, Gahan CGM, Marchesi JR (2008). Functional and comparative metagenomic analysis of bile salt hydrolase activity in the human gut microbiome. *Proceedings of the National Academy of Sciences of the United States of America*.

[29] Ticho AL, Malhotra P, Dudeja PK, Gill RK, Alrefai WA (2019). Intestinal Absorption of Bile Acids in Health and Disease. *Comprehensive Physiology*.

[30] Cortes VA, Busso D, Maiz A, Arteaga A, Nervi F, Rigotti A (2014). Physiological and pathological implications of cholesterol. *Frontiers in Bioscience (Landmark Edition)*.

[31] Duane WC, Javitt NB (1999). 27-hydroxycholesterol: production rates in normal human subjects. *Journal of Lipid Research*.

[32] Jones ML, Martoni CJ, Parent M, Prakash S (2012). Cholesterol-lowering efficacy of a microencapsulated bile salt hydrolase-active Lactobacillus reuteri NCIMB 30242 yoghurt formulation in hypercholesterolaemic adults. *The British Journal of Nutrition*.

[33] Hansson GK, Robertson AKL, Söderberg-Nauclér C (2006). Inflammation and atherosclerosis. *Annual Review of Pathology*.

[34] Ding L, Chang M, Guo Y, Zhang L, Xue C, Yanagita T (2018). Trimethylamine-N-oxide (TMAO)-induced atherosclerosis is associated with bile acid metabolism. *Lipids in Health and Disease*.

[35] Wu B, Tan L, Wang W, Feng X, Yan D (2022). Imidazole Propionate is Increased in Diabetes and Associated with Stool Consistency. *Diabetes, Metabolic Syndrome and Obesity: Targets and Therapy*.

[36] Jacobs J, Braun J (2014). Host genes and their effect on the intestinal microbiome garden. *Genome Medicine*.

[37] Zhernakova DV, Wang D, Liu L, Andreu-Sánchez S, Zhang Y, Ruiz-Moreno AJ (2024). Host genetic regulation of human gut microbial structural variation. *Nature*.

[38] Wang J, Thingholm LB, Skiecevičienė J, Rausch P, Kummen M, Hov JR (2016). Genome-wide association analysis identifies variation in vitamin D receptor and other host factors influencing the gut microbiota. *Nature Genetics*.

[39] Zhou X, Zhou X, Zhang Z, Zhu R, Lu M, Lv K (2024). Mechanism of Bile Acid in Regulating Platelet Function and Thrombotic Diseases. *Advanced Science*.

[40] Qi Z, Zhang W, Zhang P, Qu Y, Zhong H, Zhou L (2025). The gut microbiota-bile acid-TGR5 axis orchestrates platelet activation and atherothrombosis. *Nature Cardiovascular Research*.

[41] Guo C, Xie S, Chi Z, Zhang J, Liu Y, Zhang L (2016). Bile Acids Control Inflammation and Metabolic Disorder through Inhibition of NLRP3 Inflammasome. *Immunity*.

[42] Lambert G, Amar MJA, Guo G, Brewer HB, Gonzalez FJ, Sinal CJ (2003). The farnesoid X-receptor is an essential regulator of cholesterol homeostasis. *The Journal of Biological Chemistry*.

[43] Li YTY, Swales KE, Thomas GJ, Warner TD, Bishop-Bailey D (2007). Farnesoid x receptor ligands inhibit vascular smooth muscle cell inflammation and migration. *Arteriosclerosis, Thrombosis, and Vascular Biology*.

[44] Li J, Wilson A, Kuruba R, Zhang Q, Gao X, He F (2008). FXR-mediated regulation of eNOS expression in vascular endothelial cells. *Cardiovascular Research*.

[45] He F, Li J, Mu Y, Kuruba R, Ma Z, Wilson A (2006). Downregulation of endothelin-1 by farnesoid X receptor in vascular endothelial cells. *Circulation Research*.

[46] Liu TT, Wang J, Liang Y, Wu XY, Li WQ, Wang YH (2023). The level of serum total bile acid is related to atherosclerotic lesions, prognosis and gut Lactobacillus in acute coronary syndrome patients. *Annals of Medicine*.

[47] Chen ML, Zhu XH, Ran L, Lang HD, Yi L, Mi MT (2017). Trimethylamine-N-Oxide Induces Vascular Inflammation by Activating the NLRP3 Inflammasome Through the SIRT3-SOD2-mtROS Signaling Pathway. *Journal of the American Heart Association*.

[48] Yin Y, Pastrana JL, Li X, Huang X, Mallilankaraman K, Choi ET (2013). Inflammasomes: sensors of metabolic stresses for vascular inflammation. *Frontiers in Bioscience (Landmark Edition)*.

[49] Kawamata Y, Fujii R, Hosoya M, Harada M, Yoshida H, Miwa M (2003). A G protein-coupled receptor responsive to bile acids. *The Journal of Biological Chemistry*.

[50] Huber RM, Murphy K, Miao B, Link JR, Cunningham MR, Rupar MJ (2002). Generation of multiple farnesoid-X-receptor isoforms through the use of alternative promoters. *Gene*.

[51] Holt JA, Luo G, Billin AN, Bisi J, McNeill YY, Kozarsky KF (2003). Definition of a novel growth factor-dependent signal cascade for the suppression of bile acid biosynthesis. *Genes & Development*.

[52] Inagaki T, Choi M, Moschetta A, Peng L, Cummins CL, McDonald JG (2005). Fibroblast growth factor 15 functions as an enterohepatic signal to regulate bile acid homeostasis. *Cell Metabolism*.

[53] Bishop-Bailey D, Walsh DT, Warner TD (2004). Expression and activation of the farnesoid X receptor in the vasculature. *Proceedings of the National Academy of Sciences of the United States of America*.

[54] Roessler J, Zimmermann F, Schumann P, Nageswaran V, Ramezani Rad P, Schuchardt S (2024). Modulation of the Serum Metabolome by the Short-Chain Fatty Acid Propionate: Potential Implications for Its Cholesterol-Lowering Effect. *Nutrients*.

[55] Wang Y, Jones PJH, Woollett LA, Buckley DD, Yao L, Granholm NA (2006). Effects of chenodeoxycholic acid and deoxycholic acid on cholesterol absorption and metabolism in humans. *Translational Research*.

[56] Chong Nguyen C, Duboc D, Rainteau D, Sokol H, Humbert L, Seksik P (2021). Circulating bile acids concentration is predictive of coronary artery disease in human. *Scientific Reports*.

[57] Wu ZY, Song SY, Yu CQ, Sun DJY, Pei P, Du HD (2024). Associations of plasma acylcarnitine and bile acid levels with incidence of coronary heart disease in Chinese adults. *Zhonghua Yi Xue Za Zhi*.

[58] Li W, Shu S, Cheng L, Hao X, Wang L, Wu Y (2020). Fasting serum total bile acid level is associated with coronary artery disease, myocardial infarction and severity of coronary lesions. *Atherosclerosis*.

[59] Xia Y, Xu X, Guo Y, Lin C, Xu X, Zhang F (2022). Mesenchymal Stromal Cells Overexpressing Farnesoid X Receptor Exert Cardioprotective Effects Against Acute Ischemic Heart Injury by Binding Endogenous Bile Acids. *Advanced Science*.

[60] Gimbrone MA, García-Cardeña G (2016). Endothelial Cell Dysfunction and the Pathobiology of Atherosclerosis. *Circulation Research*.

[61] Zarins CK, Glagov S, Vesselinovitch D, Wissler RW (1990). Aneurysm formation in experimental atherosclerosis: relationship to plaque evolution. *Journal of Vascular Surgery*.

[62] Qin Y, Wang Y, Liu O, Jia L, Fang W, Du J (2017). Tauroursodeoxycholic Acid Attenuates Angiotensin II Induced Abdominal Aortic Aneurysm Formation in Apolipoprotein E-deficient Mice by Inhibiting Endoplasmic Reticulum Stress. *European Journal of Vascular and Endovascular Surgery*.

[63] Cole JW (2017). Large Artery Atherosclerotic Occlusive Disease.

[64] Maitrias P, Metzinger-Le Meuth V, Nader J, Reix T, Caus T, Metzinger L (2017). The Involvement of miRNA in Carotid-Related Stroke. *Arteriosclerosis, Thrombosis, and Vascular Biology*.

[65] Monteiro-Cardoso VF, Yeo XY, Bae HG, Mayan DC, Wehbe M, Lee S (2025). The bile acid chenodeoxycholic acid associates with reduced stroke in humans and mice. *Journal of Lipid Research*.

[66] Liu Y, Mao X, Li Q, Liu Y, Wu X, Chu M (2025). Increased serum total bile acid level is associated with improved prognosis of ischemic stroke. *Journal of Affective Disorders*.

[67] Watanabe M, Houten SM, Wang L, Moschetta A, Mangelsdorf DJ, Heyman RA (2004). Bile acids lower triglyceride levels via a pathway involving FXR, SHP, and SREBP-1c. *The Journal of Clinical Investigation*.

[68] Liyanage T, Ninomiya T, Wang A, Neal B, Jun M, Wong MG (2016). Effects of the Mediterranean Diet on Cardiovascular Outcomes-A Systematic Review and Meta-Analysis. *PLoS ONE*.

[69] Galié S, García-Gavilán J, Papandreou C, Camacho-Barcía L, Arcelin P, Palau-Galindo A (2021). Effects of Mediterranean Diet on plasma metabolites and their relationship with insulin resistance and gut microbiota composition in a crossover randomized clinical trial. *Clinical Nutrition*.

[70] Jing B, Gao Y, Wang L, Guo F, Jiang D, Qin S (2025). Probiotic membrane vesicles ameliorate atherosclerotic plaques by promoting lipid efflux and polarization of foamy macrophages. *Journal of Nanobiotechnology*.

[71] Jeon H, Lee D, Kim JY, Shim JJ, Lee JH (2024). Limosilactobacillus reuteri HY7503 and Its Cellular Proteins Alleviate Endothelial Dysfunction by Increasing Nitric Oxide Production and Regulating Cell Adhesion Molecule Levels. *International Journal of Molecular Sciences*.

[72] Liang X, Zhang Z, Zhou X, Lu Y, Li R, Yu Z (2020). Probiotics improved hyperlipidemia in mice induced by a high cholesterol diet via downregulating FXR. *Food & Function*.

[73] Zhao J, Cheng W, Lu H, Shan A, Zhang Q, Sun X (2022). High fiber diet attenuate the inflammation and adverse remodeling of myocardial infarction via modulation of gut microbiota and metabolites. *Frontiers in Microbiology*.

[74] Kim ES, Yoon BH, Lee SM, Choi M, Kim EH, Lee BW (2022). Fecal microbiota transplantation ameliorates atherosclerosis in mice with C1q/TNF-related protein 9 genetic deficiency. *Experimental & Molecular Medicine*.

[75] Voreades N, Kozil A, Weir TL (2014). Diet and the development of the human intestinal microbiome. *Frontiers in Microbiology*.

[76] David LA, Maurice CF, Carmody RN, Gootenberg DB, Button JE, Wolfe BE (2014). Diet rapidly and reproducibly alters the human gut microbiome. *Nature*.

[77] Just S, Mondot S, Ecker J, Wegner K, Rath E, Gau L (2018). The gut microbiota drives the impact of bile acids and fat source in diet on mouse metabolism. *Microbiome*.

[78] Gonzales E, Gerhardt MF, Fabre M, Setchell KDR, Davit-Spraul A, Vincent I (2009). Oral cholic acid for hereditary defects of primary bile acid synthesis: a safe and effective long-term therapy. *Gastroenterology*.

[79] Seethaler B, Neyrinck AM, Basrai M, Kiechle M, Delzenne NM, Bischoff SC (2025). Elucidating the effect of the Mediterranean diet on fecal bile acids and their mediating role on biomarkers of intestinal barrier function: An exploratory analysis of a randomized controlled trial. *Life Sciences*.

[80] Fitó M, Guxens M, Corella D, Sáez G, Estruch R, de la Torre R (2007). Effect of a traditional Mediterranean diet on lipoprotein oxidation: a randomized controlled trial. *Archives of Internal Medicine*.

[81] Delgado-Lista J, Alcala-Diaz JF, Torres-Peña JD, Quintana-Navarro GM, Fuentes F, Garcia-Rios A (2022). Long-term secondary prevention of cardiovascular disease with a Mediterranean diet and a low-fat diet (CORDIOPREV): a randomised controlled trial. *Lancet*.

[82] Almevall AD, Wennberg P, Liv P, Nyman E, Lindvall K, Norberg M (2025). Midlife Mediterranean Diet is Associated with Subclinical Carotid Atherosclerosis in Late Midlife. *European Journal of Preventive Cardiology*.

[83] Ridlon JM, Harris SC, Bhowmik S, Kang DJ, Hylemon PB (2016). Consequences of bile salt biotransformations by intestinal bacteria. *Gut Microbes*.

[84] Chambers ES, Preston T, Frost G, Morrison DJ (2018). Role of Gut Microbiota-Generated Short-Chain Fatty Acids in Metabolic and Cardiovascular Health. *Current Nutrition Reports*.

[85] Kawabata K, Yoshioka Y, Terao J (2019). Role of Intestinal Microbiota in the Bioavailability and Physiological Functions of Dietary Polyphenols. *Molecules*.

[86] Karlsson C, Ahrné S, Molin G, Berggren A, Palmquist I, Fredrikson GN (2010). Probiotic therapy to men with incipient arteriosclerosis initiates increased bacterial diversity in colon: a randomized controlled trial. *Atherosclerosis*.

[87] Khongrum J, Yingthongchai P, Boonyapranai K, Wongtanasarasin W, Aobchecy P, Tateing S (2023). Safety and Effects of Lactobacillus paracasei TISTR 2593 Supplementation on Improving Cholesterol Metabolism and Atherosclerosis-Related Parameters in Subjects with Hypercholesterolemia: A Randomized, Double-Blind, Placebo-Controlled Clinical Trial. *Nutrients*.

[88] Gibson GR, Roberfroid MB (1995). Dietary modulation of the human colonic microbiota: introducing the concept of prebiotics. *The Journal of Nutrition*.

[89] Everard A, Lazarevic V, Derrien M, Girard M, Muccioli GG, Neyrinck AM (2011). Responses of gut microbiota and glucose and lipid metabolism to prebiotics in genetic obese and diet-induced leptin-resistant mice. *Diabetes*.

[90] Johnson LP, Walton GE, Psichas A, Frost GS, Gibson GR, Barraclough TG (2015). Prebiotics Modulate the Effects of Antibiotics on Gut Microbial Diversity and Functioning in Vitro. *Nutrients*.

[91] Hoving LR, Katiraei S, Pronk A, Heijink M, Vonk KKD, Amghar-El Bouazzaoui F (2018). The prebiotic inulin modulates gut microbiota but does not ameliorate atherosclerosis in hypercholesterolemic APOE*3-Leiden.CETP mice. *Scientific Reports*.

[92] Hoving LR, de Vries MR, de Jong RCM, Katiraei S, Pronk A, Quax PHA (2018). The Prebiotic Inulin Aggravates Accelerated Atherosclerosis in Hypercholesterolemic APOE*3-Leiden Mice. *Nutrients*.

[93] Canfora EE, Jocken JW, Blaak EE (2015). Short-chain fatty acids in control of body weight and insulin sensitivity. *Nature Reviews. Nature Reviews*.

[94] Vulevic J, Juric A, Walton GE, Claus SP, Tzortzis G, Toward RE (2015). Influence of galacto-oligosaccharide mixture (B-GOS) on gut microbiota, immune parameters and metabonomics in elderly persons. *The British Journal of Nutrition*.

[95] Zellmer C, De Wolfe TJ, Van Hoof S, Blakney R, Safdar N (2016). Patient Perspectives on Fecal Microbiota Transplantation for Clostridium Difficile Infection. *Infectious Diseases and Therapy*.

[96] Correale J, Hohlfeld R, Baranzini SE (2022). The role of the gut microbiota in multiple sclerosis. *Nature Reviews. Neurology*.

[97] Wei J, Chen J, Fang X, Liu T, Yuan Y, Zhang J (2023). Protocol for the safety and efficacy of fecal microbiota transplantation liquid in children with autism spectrum disorder: a randomized controlled study. *Frontiers in Microbiology*.

[98] Guirro M, Costa A, Gual-Grau A, Herrero P, Torrell H, Canela N (2019). Effects from diet-induced gut microbiota dysbiosis and obesity can be ameliorated by fecal microbiota transplantation: A multiomics approach. *PLoS ONE*.

[99] Zhang PP, Li LL, Han X, Li QW, Zhang XH, Liu JJ (2020). Fecal microbiota transplantation improves metabolism and gut microbiome composition in db/db mice. *Acta Pharmacologica Sinica*.

[100] Vrieze A, Van Nood E, Holleman F, Salojärvi J, Kootte RS, Bartelsman JFWM (2012). Transfer of intestinal microbiota from lean donors increases insulin sensitivity in individuals with metabolic syndrome. *Gastroenterology*.

[101] Kootte RS, Levin E, Salojärvi J, Smits LP, Hartstra AV, Udayappan SD (2017). Improvement of Insulin Sensitivity after Lean Donor Feces in Metabolic Syndrome Is Driven by Baseline Intestinal Microbiota Composition. *Cell Metabolism*.

[102] Bustamante JM, Dawson T, Loeffler C, Marfori Z, Marchesi JR, Mullish BH (2022). Impact of Fecal Microbiota Transplantation on Gut Bacterial Bile Acid Metabolism in Humans. *Nutrients*.

[103] Paramsothy S, Paramsothy R, Rubin DT, Kamm MA, Kaakoush NO, Mitchell HM (2017). Faecal Microbiota Transplantation for Inflammatory Bowel Disease: A Systematic Review and Meta-analysis. *Journal of Crohn’s & Colitis*.

[104] Mao X, Larsen SB, Zachariassen LSF, Brunse A, Adamberg S, Mejia JLC (2024). Transfer of modified gut viromes improves symptoms associated with metabolic syndrome in obese male mice. *Nature Communications*.

[105] Allegretti JR, Kassam Z, Osman M, Budree S, Fischer M, Kelly CR (2018). The 5D framework: a clinical primer for fecal microbiota transplantation to treat Clostridium difficile infection. *Gastrointestinal Endoscopy*.

